# A Retrospective Analysis Comparing VATS Cost Discrepancies and Outcomes in Primary Lung Cancer vs. Second Primary Lung Cancer Patients

**DOI:** 10.3390/healthcare11121745

**Published:** 2023-06-14

**Authors:** Bogdan Cosmin Tanase, Alin Ionut Burlacu, Claudiu Eduard Nistor, Teodor Horvat, Cristian Oancea, Monica Marc, Emanuela Tudorache, Tudor Mateescu, Diana Manolescu

**Affiliations:** 1 Department of Thoracic Surgery, Oncology Institute “Alexandru Trestioreanu” of Bucharest, Fundeni Street 252, 022328 Bucharest, Romania; bogdan-cosmin.tanase@drd.umfcd.ro (B.C.T.); ncd58@yahoo.com (C.E.N.); teodor.horvat@umfcd.ro (T.H.); 2 Center for Research and Innovation in Precision Medicine of Respiratory Diseases, “Victor Babes” University of Medicine and Pharmacy, Eftimie Murgu Square 2, 300041 Timisoara, Romania; marc.monica@umft.ro (M.M.); tudorache_emanuela@yahoo.com (E.T.); 3 Doctoral School, “Victor Babes” University of Medicine and Pharmacy, Eftimie Murgu Square 2, 300041 Timisoara, Romania; tudor_mat@yahoo.ro; 4 Department of Radiology, “Victor Babes” University of Medicine and Pharmacy, Eftimie Murgu Square 2, 300041 Timisoara, Romania; dmanolescu@umft.ro

**Keywords:** lung cancer, pulmonary disease, video-assisted thoracoscopic surgery

## Abstract

This study aimed to compare the outcomes and cost differences between primary lung cancer (PLC) and second primary lung cancer (SPLC) patients who underwent video-assisted thoracoscopic surgery (VATS). This was a retrospective analysis of 124 patients with lung cancer stages I, II, and III who underwent VATS between January 2018 and January 2023. The patients were divided into two groups based on their cancer status that was matched by age and gender: the PLC group (*n* = 62) and the SPLC group (*n* = 62). The results showed that there was no significant difference in the clinical characteristics between the 2 groups, except for the Charlson Comorbidity Index (CCI), with a score above 3 in 62.9% of PLC patients and 80.6% among SPLC patients (*p* = 0.028). Regarding the surgical outcomes, the operative time for the VATS intervention was significantly higher in the SPLC group, with a median of 300 min, compared with 260 min in the PLC group (*p* = 0.001), varying by the cancer staging as well. The average duration of hospitalization was significantly longer before and after surgery among patients with SPLC (6.1 days after surgery), compared with 4.2 days after surgery in the PLC group (0.006). Regarding the cost analysis, the total hospitalization cost was significantly higher in the SPLC group (15,400 RON vs. 12,800 RON; *p* = 0.007). Lastly, there was a significant difference in the survival probability between the two patient groups (log-rank *p*-value = 0.038). The 2-year survival was 41.9% among PLC patients and only 24.2% among those with SPLC. At the 5-year follow-up, there were only 1.6% survivors in the SPLC group, compared with 11.3% in the PLC group (*p*-value = 0.028). In conclusion, this study found that VATS is a safe and effective surgical approach for both PLC and SPLC patients. However, SPLC patients have a higher VATS operating time and require more healthcare resources than PLC patients, resulting in higher hospitalization costs. These findings suggest that careful pre-operative evaluation and individualized surgical planning are necessary to optimize the outcomes and cost-effectiveness of VATS for lung cancer patients. Nevertheless, the 5-year survival remains very low and concerning.

## 1. Introduction

Lung cancer is a leading cause of cancer-related mortality worldwide, although the incidence varies widely across the world, with higher rates in developed countries where smoking prevalence is high [1,2,3,4]. In terms of outcomes, lung cancer has a poor prognosis, with a 5-year survival rate of around 15–20%, although the survival rate is highly dependent on the stage at which the cancer is diagnosed [5]. Early-stage lung cancer has a better prognosis than advanced-stage lung cancer. Unfortunately, many cases of lung cancer are diagnosed at an advanced stage, which reduces the chance of successful treatment and long-term survival [6,7].

Lung cancer may arise as a second primary lung cancer (SPLC) that can develop from earlier cancer therapy or contact with oncogenic substances, with an estimated risk of 1–2% per patient per year [8,9,10]. The type and dosage of cancer therapy as well as patient-related characteristics, including age, gender, and smoking history, all have an impact on the epidemiology of lung cancer as a secondary malignancy [11]. Radiation therapy, chemotherapy, and targeted therapy are a few popular cancer therapies that increase the risk of secondary lung cancer [12], particularly in individuals who have had radiation treatment to the chest region, which has been associated with an elevated risk of subsequent lung cancer development [13]. Lung metastases, on the other hand, are distinct from second primary lung cancer and affect, on average, 30% of all cancer patients [14].

The choice of treatment depends on the stage and type of cancer, as well as the patient’s overall health and preferences [15,16]. Among the surgical methods used in lung cancer treatment are video-assisted thoracoscopic surgery (VATS) [17]. The first VATS lobectomy was performed in France in 1992 [18]. While over time it has been shown to be a safe and effective treatment option for early-stage lung cancer, with comparable long-term outcomes to open surgery, it has recently also been used to treat more advanced stages of lung cancer, including metastatic disease [19,20,21]. As a minimally invasive surgical technique, VATS offers numerous benefits for patients with lung cancer, such as less pain, shorter hospitalization, faster recovery times, lower rates of complications, and improved survival rates [22,23]. These outcomes are hypothesized to reduce the cost burden of lung cancer treatment and improve patient satisfaction, overall wellbeing, and health-related quality of life [24,25,26,27].

As such, VATS is an important intervention that can improve the quality of life for patients with lung cancer and contribute to more efficient and effective healthcare delivery. Therefore, the primary objective of the current study was to observe the difference in pre-interventional and post-interventional outcomes between patients with primary lung cancer and second primary lung cancer. The secondary aim of this study was to determine the cost differences between VATS surgery and hospitalization among these two patient groups.

## 2. Materials and Methods

### 2.1. Study Design and Ethical Considerations

A multicentric retrospective cohort study was designed at the Clinical Hospital for Infectious Diseases and Pneumology in Timisoara and the Oncology Institute “Alexandru Trestioreanu” of Bucharest, Romania. The study was conducted according to the guidelines of the Declaration of Helsinki and given approval number 23 on 6 February 2023. The data collection period was set between January 2018 and January 2023, while the researchers gathered data from the hospital databases and the associated patients’ paper records, where all treatments, procedures, and laboratory analyses were registered.

All patients included in the study had their initial diagnosis in 2018, and a follow-up period until January 2023 or until death. The inclusion criteria for the PLC group comprised the following particularities: (1) patients required to be at least 18 years old; (2) patients must have a histological diagnosis of primary lung cancer; (3) no previous history of lung cancer. The inclusion criteria in the SPLC group comprised: (1) adult patients older than 18 years with confirmed histology of lung cancer; (2) a previous history of lung cancer that was previously treated or in remission. The exclusion criteria comprised: (1) incomplete medical records; (2) lack of consent identified from the personal paper records; (3) patients were also excluded if the diagnosis of lung cancer was secondary to a pre-existing malignancy as a secondary determination; (4) patients with TNM stage IV, pre-operative chemotherapy or radiation therapy, metastatic and locally invasive lung cancer, and patients who underwent open surgery instead of VATS. After the patient selection process, the entire cohort was split into a group of patients with primary lung cancer who underwent VATS and a group of patients with second primary lung cancer who underwent VATS. The two groups were case-matched 1:1 based on age and gender.

### 2.2. Procedures and Variables

The study cohort was identified based on the inclusion and exclusion criteria. The inclusion criteria were defined based on the research question, and the exclusion criteria were set to avoid any confounding factors. Data were extracted from the medical records of the study cohort. Standardized data extraction forms were used, and data included demographics, clinical characteristics, treatment history, and follow-up data. Quality control checks were performed after data extraction to ensure that the data was accurate and complete, which involved checking for missing data, outliers, and inconsistencies. The extracted data was stored in a spreadsheet for further analysis. The variables considered to be included in this study comprised: age, age range, gender, area of residence, smoking status, pack-year smoking history, exposure to respiratory hazards, blood type, Charlson Comorbidity Index [28], forced expiratory volume, ejection fraction of the left ventricle, lung cancer histology, lung cancer localization, the lobe involved, tumor node metastasis (TNM) cancer staging, blood loss during the intervention, operative time, lymphadenectomy, drainage volume during the intervention, total intravenous fluids during the intervention, local anesthesia, intra-operative diuresis, surgical site infection, surgical site seroma, air leak, days of hospitalization, days in the intensive care unit (ICU), Clavien-Dindo score [29], local tumoral invasion after surgery, distant invasion after surgery, total expenses, and patients’ survival.

The risk of bias assessment was performed to evaluate the quality of the study. The assessment involved evaluating the study’s internal validity, and it was done using the Cochrane Risk of Bias tool or the Newcastle-Ottawa Scale [30]. The assessment criteria included selection bias, information bias, and confounding factors. The sample size calculation was performed to determine the number of participants needed to achieve statistical power. The calculation involved considering the effect size, level of significance, and power of the study. The sample size calculation was based on the following factors: (1) the expected difference in the outcome between the two groups; (2) the level of statistical significance of 0.05; (3) the power of the study of 0.80; and (4) the estimated standard deviation of the outcome variable. Patients’ lung cancer staging was assessed using the AJCC guidelines and the eighth edition of the TNM staging system [31], while the VATS and open surgery were performed according to existing protocols [32]. Due to the retrospective nature of the study, more than one operator was involved in the treatment of these patients.

### 2.3. Statistical Analysis

GraphPad Prism for Microsoft Windows, version 6.0, was used to conduct the statistical analysis (GraphPad Software, San Diego, CA, USA). The Kolmogorov-Smirnov test was used to assess the normality of the data. The mean value, which represents central tendency, and the standard deviation, which measures dispersion, were used to represent normally distributed data. Student’s *t*-test was used to examine the difference in means between the two comparison groups. The median and interquartile range (IQR) were used to characterize non-normally distributed data, presented in box plots, while the Mann-Whitney u-test was used to compare these variables. Considering the frequency assumption for the Chi-square test was not fulfilled, proportions were compared using Fisher’s exact test. A Kaplan-Meier curve was plotted to observe the survival probability. A *p*-value below 0.05 was regarded as statistically significant.

## 3. Results

### 3.1. Background Analysis

A total of 124 patients were eligible for inclusion in the current study, out of which 62 were diagnosed with PLC and underwent a VATS intervention, and the other half were diagnosed with SPLC and underwent a VATS procedure as well. The mean age in the primary group was 62.1 years compared to 63.7 in the second group, while patients’ age ranged from 37 to 84 years. The majority of patients were women (53.2% in the primary group vs. 58.1% in the second group). Almost half of all individuals analyzed had a history of smoking or were current smokers, with a median of 30.5 pack-years in the primary group, respectively, 32.5 pack-years in the second group, without any statistically significant differences. Additionally, the majority of patients with primary lung cancer in this cohort had blood type A. There was a significant difference between the two study groups regarding their comorbidity status. The CCI score was above 3 in 62.9% of patients from the primaryPLC group and 80.6% in the SPLC group, respectively, as described in Table 1.

### 3.2. Clinical and Oncological Features

**Figure 1:**
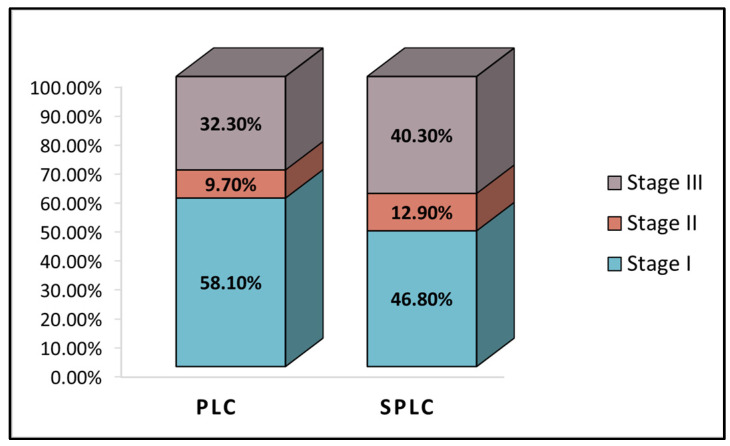
TNM cancer staging in the study cohort; PLC—Primary Lung Cancer; SPLC—Second Primary Lung Cancer.

The pre-operative evaluation identified an FEV1% average value of 79.7 in the primary group, compared to 79.8 in the second group, as presented in Table 2. The left ventricle ejection fraction was 57.2% in those from the PLC group compared with 57.7% in the SPLC group. There were 83.9% NSCLC cases in the primary group and 72.6% in the SPLC group, respectively. The most involved anatomical region was the right lung (63.8% in the entire cohort), while particularly the left upper lobe was the most commonly affected area in the PLC group (29.0%), respectively, the right upper lobe in the secondary group 32.3%, without statistical significance between groups. Lastly, the TNM staging, described in Figure 1, identified 32.3% of patients with Stage III PLC who underwent VATS, compared with 40.3% of Stage III in the SPLC group.

### 3.3. Surgical Intervention and Outcomes

**Figure 2:**
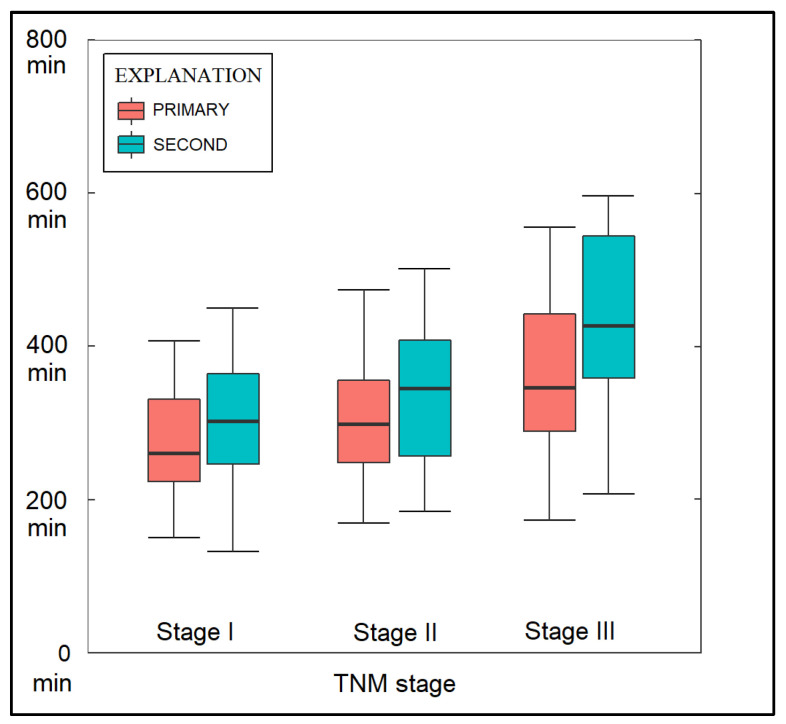
Operative time measured in minutes; min—minutes; PLC—Primary Lung Cancer; SPLC—Second Primary Lung Cancer.

The volume of blood loss during the surgical intervention was significantly higher in the SPLC group, with a proportion of 21.0% of patients losing more than 200 mL of blood, compared to only 9.7% of patients in the PLC group (*p*-value = 0.028), as described in Table 3. As seen in Figure 2, the operative time for the VATS intervention was significantly higher in the SPLC group, with a median of 300 min, compared with 260 min for VATS in the PLC (*p*-value = 0.001). It was also observed that the median operative time increased from 260 min in patients with stage I in the PLC group to 320 min in those with stage III PLC. Among those with stage I SPLC, the operative time for VATS had a median of 290 min, while in stage III SPLC, it increased to 380 min. Interestingly, the total IV fluid necessitated by patients from the PLC group was significantly higher than in the SPLC group (2370 mL vs. 2161 mL, *p*-value = 0.040).

**Figure 3:**
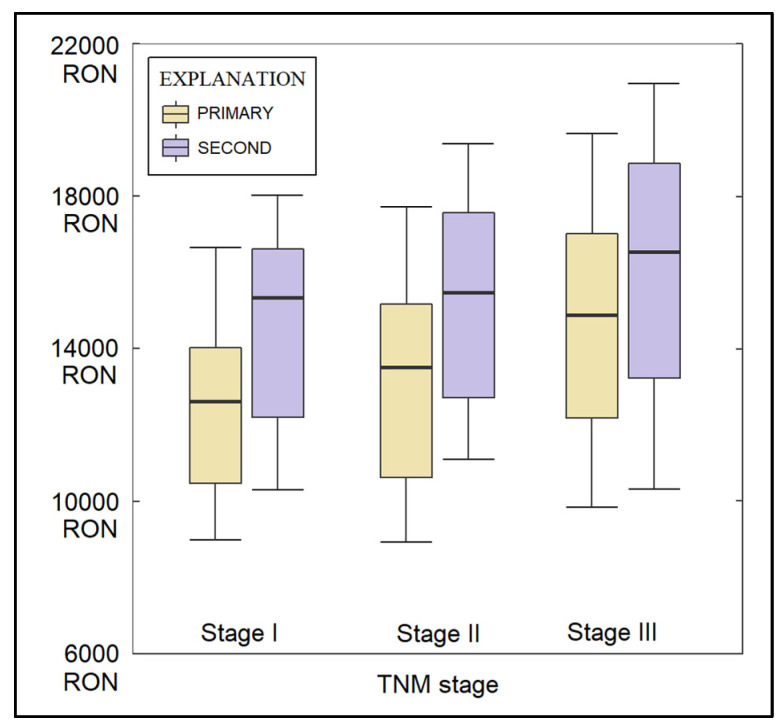
Total costs measures in RON; RON—Romanian currency; PLC—Primary Lung Cancer; SPLC—Second Primary Lung Cancer.

Regarding the post-surgical complications, there were no significant differences regarding the air leaks, surgical site infections, and the development of seromas. The average duration of hospitalization was significantly longer both before and after surgery among patients with SPLC (6.1 days after surgery, compared with 4.2 days after surgery in the PLC group, *p*-value = 0.004). Similarly, the total expenses were significantly higher among patients with SPLC (15,400 RON vs. 12,800 RON in the primary group, *p*-value = 0.007), as seen in Figure 3. Post-surgical complications measured by the Clavien-Dindo scale identified 82.3% of patients with a score of I in the PLC group and 66.1% with a score of I in the SPLC group (*p*-value = 0.040). Similarly, the proportion of patients with distant tumoral invasion was significantly higher among patients with SPLC (12.9% vs. 3.2%, *p*-value = 0.007).

**Figure 4:**
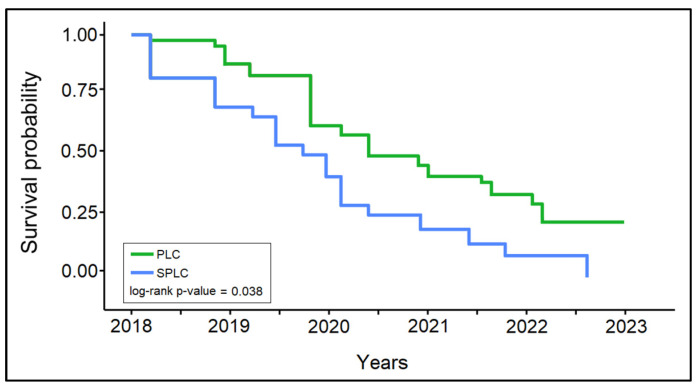
Kaplan-Meier survival curve; PLC—Primary Lung Cancer; SPLC—Second Primary Lung Cancer.

The survival analysis of patients with PLC and SPLC, presented in Table 4 and Figure 4, identified a significant difference in the survival probability between the two patient groups (log-rank *p*-value = 0.038). The 1-year survival did not show significant differences between the PLC group (82.3%) and the SPLC group (74.2%). However, after 2 years, the difference became significant, where 41.9% of patients from the PLC group survived, compared to only 24.2% in the SPLC group (*p*-value = 0.035). Lastly, at the 5-year follow-up, there were only 1.6% survivors in the SPLC group, compared with 11.3% in the PLC group (*p*-value = 0.028).

## 4. Discussion

### 4.1. Literature Findings

The results of this study suggest that there are no significant short-term differences in VATS outcomes between primary lung cancer and second primary lung cancer patients. This is consistent with previous studies that have shown that VATS is a safe and effective treatment option for both primary and second primary lung cancer. However, the cost of VATS is significantly higher for SPLC patients, as they may have a higher burden of comorbidities or may require more extensive pre-operative testing, which could increase the cost of VATS. Furthermore, SPLC patients may require more extensive surgery, or a longer hospital stay, which could also contribute to the higher costs.

Although the cost differences of VATS between PLC and SPLC patients is of important debate in the current study, the literature is very limited. Thus, we might consider the literature that compares costs of VATS with open surgery, as reference to our findings. In a Chinese study performed at a provincial referral cancer center in 2021, researchers compared cost-related clinical outcomes and healthcare costs of VATS lobectomy and open lobectomy for lung cancer patients [33]. A total of 376 patients were selected for analysis, and it was observed that VATS lobectomy group experienced lower blood transfusion rates (2.1% vs. 3.1%), lower lung infection rates (21.2% vs. 39.8%), and shorter post-operative length of stay (9.4 days vs. 10.8 days) compared to the open lobectomy group. These findings are in accordance with our results, where patients had a total hospitalization time of approximately 10 days. Total hospitalization costs were similar between the 2 groups (RMB 84,398 vs. RMB 81,964); however, total non-surgery costs were significantly lower in the VATS lobectomy group (RMB 41,948 vs. RMB 45,752). Therefore, although VATS surgery can be more expensive as procedure, the factors associated with non-surgical costs determine a significant reduction of total costs.

Similarly, in another study by Swanson et al., the researchers compared the hospital costs and perioperative outcomes of 3961 patients who underwent lobectomy for cancer by either VATS or open thoracotomy in the United States [34]. The study found that hospital costs were lower for VATS procedures, at an average of USD 20,316, than open procedures (USD 21,016), with costs decreasing further as the surgeon’s experience increased [35]. length of stay was shorter for VATS patients (6.2 days) compared to open surgery patients (7.8 days), and the risk of adverse events was significantly lower in the VATS group (odds ratio of 1.22). However, surgery duration was shorter for open procedures (3.7 h) than for VATS procedures (4.1 h).

Similarly, one randomized trial was carried out by Pompeo et al. in 2007 [36]. During a thoracic epidural anesthesia, VATS bullectomy and pleurodesis were performed on 43 patients who had spontaneous pneumothorax. Their findings demonstrated that the VATS procedure was both safe and effective, in addition to resulting in a shorter hospital stay and lower overall costs. Another study investigated the relationship between surgeon experience and cost in VATS and open lobectomy procedures, given the 1.68-day reduction in length of stay and decrease in adverse events with VATS [37]. A significant association between surgeon experience and cost was found for VATS, with average costs ranging from USD 22,000 for low-volume surgeons to approximately USD 18,000 for high-volume surgeons. In contrast, there was no significant difference in cost for open lobectomies based on surgeon experience, with both levels estimated at USD 21,000. These findings are in accordance with the study by Fang et al. [38], who also reported average costs around USD 20,000, and suggested that the economic impact is greater as the surgeon’s experience with VATS increases. These findings from the US study, performed in 2007, are in contrast with our reported costs, where we averaged approximately USD 3000 at the 2022 conversion rate, while also considering the inflation between these 15 years.

In Italy, between January 2004 and December 2006, 346 patients underwent pulmonary lobectomy for mainly stage I or II lung cancer, with 93 undergoing VATS lobectomy and 253 undergoing thoracotomy [39]. The mean theater cost for VATS lobectomy was significantly higher at EUR 2533 compared to EUR 1280 for thoracotomy lobectomy. However, VATS lobectomy had a lower mean high dependency unit cost (EUR 1713) and hospital stay cost (EUR 3776) than thoracotomy lobectomy, with respective costs of EUR 2571 and EUR 4325, which are similar to the VATS costs from our Romanian surgical center. The overall cost of VATS lobectomy (EUR 8023) was lower than that of open lobectomy (EUR 8178). Nevertheless, it is clear that the overall cost varies significantly by region, date of study, surgeon performance, availability of resources, and the currency used in the specific country. Moreover, in the recent years the COVID-19 pandemic greatly impacted the healthcare systems worldwide, decreasing the availability for surgical intervention during the pandemic peaks, and increasing the costs of hospitalization, as shown in a study conducted in Italy [40].

Regarding the results and outcomes of patients with primary lung cancer and second primary lung cancer, one meta-analysis highlighted VATS lobectomy had significantly better 5-year overall survival rates compared to open lobectomy, although no statistically significant difference was observed in 1.3-year overall survival between the two groups [41]. Nevertheless, in order for VATS lobectomy to result in improved overall survival and DFS survival, careful preoperative selection and optimization are regarded significant contributing factors [42]. The anesthesiologist’s role is particularly vital in VATS, which requires single-lung ventilation, often necessitating the use of a double-lumen tube or bronchial blocker, both of which require expertise in their placement and management. Appropriate anesthetic management is also essential to ensure optimal surgical conditions, prevent intraoperative complications, and promote faster postoperative recovery. The reduced invasiveness of VATS and improvements in surgical techniques were cited as possible explanations. VATS lobectomy was associated with better preserved cellular immunity and less immunosuppression during the immediate postoperative period, which may have contributed to improved long-term survival and reduced systemic recurrence. Additionally, a longer operative duration could have impacted long-term clinical outcomes.

A study conducted in Japan on 1340 patients with PLC and metachronous SPLC in which 75% underwent VATS, observed a rate of grade II or higher postoperative complications of 24.0% and 22.0%, respectively [43]. The authors reported an average operative time of 171 min for PLC patients, and 203 min for SPLC patients, which is significantly shorter than the median time for surgery reported in our study (260 min for PLC vs. 300 min for SPLC). However, the operative time range in the study by Muranishi et al. was as high as 360 min for PLC and 568 min for SPLC. Additionally, no cost-analysis was performed in the Japanese study, which could have served as reference.

Moreover, the 5-year overall survival rates were 68.7% and 83.0% in the PLC and the SPLC groups [43], respectively, which are significantly higher than the survival rates in our study (11.3% for PLC patients and 1.6% for SPLC patients, respectively). It is important to acknowledge that variations in survival rates can stem from population differences, including genetic, lifestyle, and environmental factors. The discrepancies might be attributed to differences in the genetic profiles, prevalence of comorbidities, or lifestyle factors such as smoking habits, between the populations in our study and the Japanese study. Moreover, it is also important to consider statistical factors such as the cohort size in each study, which can influence survival outcomes, since the sample size in our study was considerably smaller.

Other authors demonstrated that secondary VATS was both feasible and safe for patients who had previously undergone pulmonary resection. The median duration for secondary surgery was 120 min, and the median blood loss was 50 mL [44]. Nevertheless, several complications and observations were found in patients with a secondary intervention for lung cancer using VATS. Several patients required conversion to thoracotomy due to severe pleural adhesions, while two patients needed chest tube reinsertion due to pneumothorax, but no patients died within 30 days of the procedure. The study also found that high preoperative Charlson Comorbidity Index and severe pleural adhesions were independent risk factors for complications in SPLC patients. Nevertheless, in the current study, the CCI was taken into consideration for pre-operability evaluation of patients, as well as the FEV1% and the ejection fraction of the left ventricle. However, other complementary assessments, such as the cardiopulmonary exercise test, diffusing capacity for carbon monoxide, peak oxygen consumption, or FEV/FVC, were proven to be more accurate evaluation tools and predictors for complications [45].

Pleural adhesions, which result from the body’s natural repair process after the initial operation, can lead to unclear tissue boundaries, increased intraoperative bleeding, and the possibility of injury to vital organs due to a more challenging dissection of patients with SPLC. In this study, 10 patients (14.3%) required conversion to thoracotomy in secondary surgery, which was an improvement over the previously reported 20% conversion rate. The postoperative complication rate in this study was also lower compared to prior studies, with rates of 29.0%, 34.3%, and 36.5% reported in other research [46,47]. Moreover, when the initial and secondary tumors are located on the same side, the dissection can be particularly challenging due to the dense fibrous adhesions that can form after the initial surgery. This increased complexity of the VATS intervention can lead to higher costs due to the additional time and resources needed to carefully navigate the fibrotic tissue, increased risk of conversion to thoracotomy, or an increased risk of postoperative complications such as prolonged air leak or infection [48]. Thus, the use of adhesion prevention strategies can be beneficial in these cases [49]. Although our retrospective study lacks information regarding the extension and severity of adhesions found during surgery, the antiadhesion strategies performed during VATS in the current study involved limited dissection of the surrounding tissues, saline lavage of the pleural space, and application of a hyaluronic acid-based substance Hyalobarrier Gel Endo (Anika Therapeutics, Bedford, MA, USA).

Air leakage was identified as the most common complication of secondary pulmonary resection, primarily caused by intraoperative adhesion [50]. The study aimed to provide guidance for future research by defining and analyzing the degree of pleural adhesion. In addition to careful treatment of pleural adhesions, the authors suggested that carefully checking the area and repairing lung tissue with prolene sutures could effectively prevent air leakage. Other techniques, such as using free pericardial fat pads as sealants or employing the TachoSil technique, have also been reported to reduce the duration of persistent postoperative air leakage [51].

### 4.2. Study Strengths and Limitations

Due to the lack of randomization in retrospective studies, it was opted here to match the two patient groups by their background characteristics (age and gender) to control for confounding factors, since patients with second primary lung cancer may have different characteristics than those with primary lung cancer, that may affect the outcomes and cost differences. Nevertheless, the study may not control for all the potential confounding variables that may influence the outcomes or cost differences between the two groups, such as the CCI that was significantly higher in patients with SPLC. Another strength of this study is the long follow-up period of five years that allowed us to observe the long-term outcomes, such as survival rates and recurrence rates. One limitation is the small sample size of 124 patients in this study, which may limit the generalizability of the findings since it may not be representative of the larger population of lung cancer patients, and the results may not be applicable to patients with different characteristics or from different regions. Another limitation is the scarcity of the literature comparing cost differences between VATS in patients with PLC and SPLC, which could serve as reference for our observations. Moreover, it is important to note that the cost of VATS reported in this study may not be generalizable to other settings, as the cost of VATS may vary depending on the geographic location, hospital type, and payer mix. Therefore, further research is needed to confirm these findings in other populations.

## 5. Conclusions

In conclusion, this study provides evidence that VATS short-term outcomes are comparable between primary lung cancer and second primary lung cancer patients, although with higher operating times and hospitalization times for those with SPLC. Additionally, the cost of VATS is significantly higher for second primary lung cancer patients. Although the 1-year survival rate is similar, the 5-year survival rate is significantly lower among patients with SPLC. These findings have important implications for the management of lung cancer patients, particularly in the context of rising healthcare costs. Further research is needed to confirm these findings and to explore the factors that contribute to the cost differences between the two groups.

## Figures and Tables

**Table 1:** Background analysis.

Variables	PLC (*n* = 62)	SPLC (*n* = 62)	*p*-Value
Age (mean ± SD)	62.1 ± 9.0	63.7 ± 8.8	0.318
Age range	42–77	37–84	–
Gender (male, %)	29 (46.8%)	26 (41.9%)	0.587
Area of residence (urban, %)	52 (83.9%)	47 (75.8%)	0.263
Smoking status (yes, %)	33 (53.2%)	24 (38.7%)	0.104
Pack-year smoking (median, IQR)	30.5 (23.0–37.5)	32.5 (22.0–38.0)	0.661
Exposure to respiratory hazards (yes, %)	10 (16.1%)	7 (11.3%)	0.433
Blood type A (*n*, %)	25 (40.3%)	28 (45.2%)	0.586
CCI > 3 (*n*, %)	39 (62.9%)	50 (80.6%)	0.028

SD—Standard Deviation; IQR—Interquartile Range; CCI—Charlson Comorbidity Index; PLC—Primary Lung Cancer; SPLC—Second Primary Lung Cancer.

**Table 2:** Characteristics of lung cancer in the study cohort and pre-operative findings.

Variables	PLC (*n* = 62)	SPLC (*n* = 62)	*p*-Value
FEV1% (mean ± SD)	79.7 ± 11.0	79.8 ± 8.6	0.955
EF% (mean ± SD)	57.2 ± 2.8	57.7 ± 2.9	0.330
Cancer histology			0.127
NSCLC (*n*, %)	52 (83.9%)	45 (72.6%)	
SCLC (*n*, %)	10 (16.1%)	17 (27.4%)	
Localization			0.191
Left lung (*n*, %)	26 (41.9%)	19 (30.6%)	
Right lung (*n*, %)	36 (58.1%)	43 (69.4%)	
Lobe involved			0.858
Left upper lobe	18 (29.0%)	14 (22.6%)	
Left lower lobe	9 (14.5%)	7 (11.3%)	
Right upper lobe	16 (25.8%)	20 (32.3%)	
Right middle lobe	5 (8.1%)	6 (9.7%)	
Right lower lobe	14 (22.6%)	15 (24.2%)	
TNM staging			0.450
Stage I (all stages)	36 (58.1%)	29 (46.8%)	
Stage II (all stages)	6 (9.7%)	8 (12.9%)	
Stage III (all stages)	20 (32.3%)	25 (40.3%)	

FEV—Forced Expiratory Volume; EF—Ejection Fraction (left ventricle); NSCLC—Non-Small Cell Lung Cancer; SCLC—Small Cell Lung Cancer; TNM—Tumor, Node, Metastasis; SD—Standard Deviation; PLC—Primary Lung Cancer; SPLC—Second Primary Lung Cancer.

**Table 3:** Surgical intervention and outcomes.

Variables	PLC (*n* = 62)	SPLC (*n* = 62)	*p*-Value
Blood loss (*n*, %)			0.028
<100 mL	49 (79.0%)	48 (77.4%)	
100–200 mL	7 (11.3%)	3 (1.6%)	
>200 mL	6 (9.7%)	13 (21.0%)	
Operative time, minutes (median, IQR)	260 (223–311)	300 (248–352)	0.001
Lymphadenectomy (*n*, %)			0.278
1 group	5 (8.1%)	9 (14.5%)	
2 groups	10 (16.1%)	14 (22.6%)	
>2 groups	47 (75.8%)	39 (62.9%)	
Drainage, mL (mean ± SD)			
1st day	247.7 ± 64.9	246.8 ± 58.3	0.935
2nd day	205.0 ± 82.8	187.4 ± 73.3	0.212
Total intravenous fluids, mL (mean ± SD)	2370.9 ± 538.3	2161.3 ± 587.3	0.040
Local anesthesia (*n*, %)	48 (77.4%)	41 (66.1%)	0.162
Intra-op diuresis (mean ± SD)	1181.5 ± 371.2	1023.4 ± 362.1	0.017
Surgical site infection (*n*, %)	2 (3.2%)	4 (6.5%)	0.402
Surgical site seroma (*n*, %)	3 (4.8%)	2 (3.2%)	0.648
Air leak			
1st day post-op	5 (8.1%)	8 (12.9%)	0.379
1-week post-op	2 (3.2%)	1 (1.6%)	0.558
Intractable pain (*n*, %)	3 (4.8%)	6 (9.7%)	0.299
Reintervention (*n*, %)	1 (1.6%)	2 (3.2%)	0.559
Adjuvant treatment	40 (64.5%)	48 (77.4%)	0.113
Days of hospitalization (mean ± SD)			
Before surgery	4.2 ± 3.2	6.1 ± 4.0	0.004
After surgery	4.8 ± 2.6	6.6 ± 4.3	0.006
Days in the ICU (median, IQR)	1.0 (1.0–1.5)	1.5 (1.0–2.0)	0.162
Clavien–Dindo score I, (*n*, %)	51 (82.3%)	41 (66.1%)	0.040
Local invasion after surgery (*n*, %)	4 (6.5%)	5 (8.1%)	0.729
Distant invasion after surgery (*n*, %)	2 (3.2%)	8 (12.9%)	0.048
Total expenses, RON (median, IQR)	12,800 (9940–16,660)	15,400 (10,120–19,750)	0.007

IQR—Interquartile Range; SD—Standard Deviation; RON—Romanian currency; PLC—Primary Lung Cancer; SPLC—Second Primary Lung Cancer.

**Table 4:** Survival analysis.

Variables	PLC (*n* = 62)	SPLC (*n* = 62)	*p*-Value
1-month survival	62 (100%)	59 (95.2%)	0.079
3 months survival	60 (96.8%)	57 (91.9%)	0.243
1-year survival	51 (82.3%)	46 (74.2%)	0.276
2 years survival	37 (58.1%)	25 (40.3%)	0.031
3 years survival	26 (41.9%)	15 (24.2%)	0.035
5 years survival	7 (11.3%)	1 (1.6%)	0.028

PLC—Primary Lung Cancer; SPLC—Second Primary Lung Cancer.

## Data Availability

Data are available on request.
